# Intramuscular Hemangioma of the Tongue: Imaging Features of a Rare Entity

**DOI:** 10.7759/cureus.101540

**Published:** 2026-01-14

**Authors:** Sagar V Gund, Kajal Mitra, Suresh Phatak, Pranit B Pantawane, Akanksha P Kalwaghe

**Affiliations:** 1 Radiology, Narendra Kumar Prasadrao (NKP) Salve Institute of Medical Sciences and Research Center, Nagpur, IND

**Keywords:** flow voids, histopathological, intramuscular hemangioma, magnetic resonance imaging, tongue

## Abstract

Intramuscular hemangioma (IMH) is a rare benign vascular tumor arising from skeletal muscle. Head and neck involvement is rare, with tongue muscles being extremely rare. IMHs present as a slow-growing mass. They are painless; however, sometimes, they may cause speech difficulties or bleeding. Imaging is essential for diagnosis and surgical planning. Magnetic resonance imaging (MRI) provides superior soft tissue characterization. This is a case report of a 25-year-old woman with a complaint of a painless swelling over the dorsum of the tongue. MRI revealed a well-circumscribed, lobulated lesion appearing hyperintense on T2-weighted imaging (T2WI), with few flow voids, showing intense post-contrast enhancement. Complete transoral excision was performed, and histopathology confirmed the lesion as an intramuscular cavernous hemangioma. This case focuses on the importance of multimodality imaging and radio-pathological correlation in the diagnosis and management of rare intramuscular tongue hemangiomas.

## Introduction

Hemangiomas are non-malignant vascular tumors defined by abnormal proliferation of blood vessels. They frequently occur in the head and neck region. However, hemangiomas arising from skeletal muscle, termed intramuscular hemangiomas, are rare entities, representing less than 1% of all hemangiomas [1]. The tongue is an unusual site for intramuscular hemangioma because of its complex musculature and deep-seated location, which makes it difficult to diagnose [2]. Clinically, the lesion commonly presents as a painless and slow-growing mass with intact overlying mucosa. If large, then it may be associated with speech disturbance and dysphagia, and may bleed in advanced cases; therefore, careful surgical resection is necessary [3]. As intramuscular hemangiomas of the tongue are rare and have a nonspecific presentation, they may frequently be misdiagnosed as cystic lesions or other benign soft tissue lesions, such as lymphatic malformations, other vascular tumors, venous malformations, and benign soft tissue lesions, thus highlighting the importance of radiological evaluation and histopathological confirmation [1,4,5].

## Case presentation

A 25-year-old female patient came with chief complaints of difficulty talking and chewing food.

**Figure 1:**
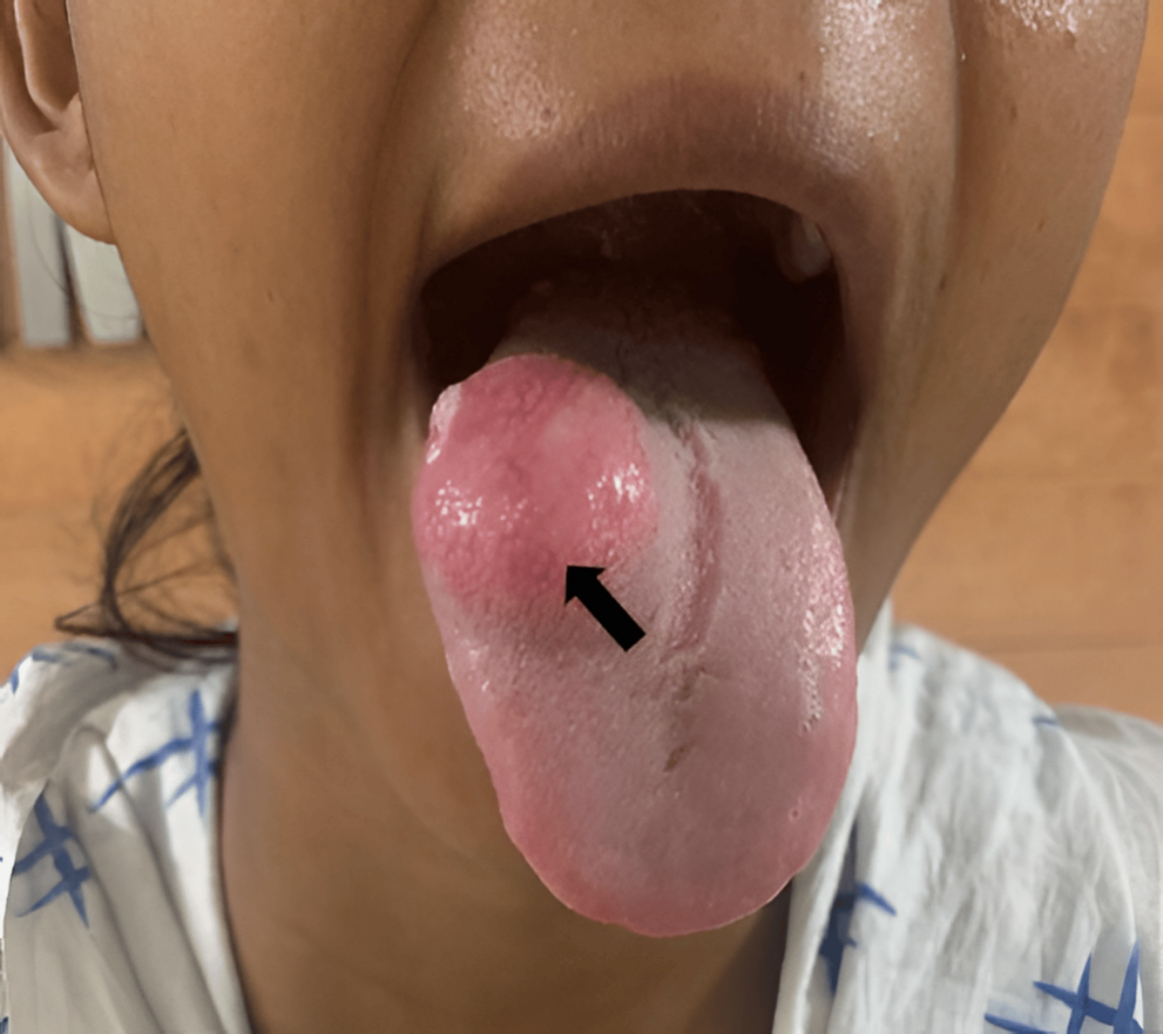
Reddish-pink colored lesion noted over the dorsal aspect of the tongue on the right side

A soft-to-firm, non-tender, reddish-pink colored lesion was noted over the dorsal aspect of the tongue on the right side in intraoral examination (Figure 1). On application of pressure, blanching occurred, thus suggesting a vascular lesion. This lesion showed a gradual increase in size since birth. Occasional bleeding from the lesion was present. Cervical lymph nodes were not palpable. Magnetic resonance imaging (MRI) showed a well-defined altered signal intensity lesion of size approximately 2.7 × 1.9 × 2.5 cm (anterior-posterior (AP) × transverse (TR) × craniocaudal (CC) dimensions, respectively). It showed a T1-weighted imaging (T1WI) isointense and T2/short-tau inversion recovery (STIR) hyperintense lesion involving the genioglossus and hyoglossus muscles of the tongue on the dorsal aspect, containing flow voids and homogeneous post-contrast enhancement. No adjacent bone involvement or extrinsic muscle infiltration was noted. These findings were consistent with intramuscular hemangioma.

**Figure 2:**
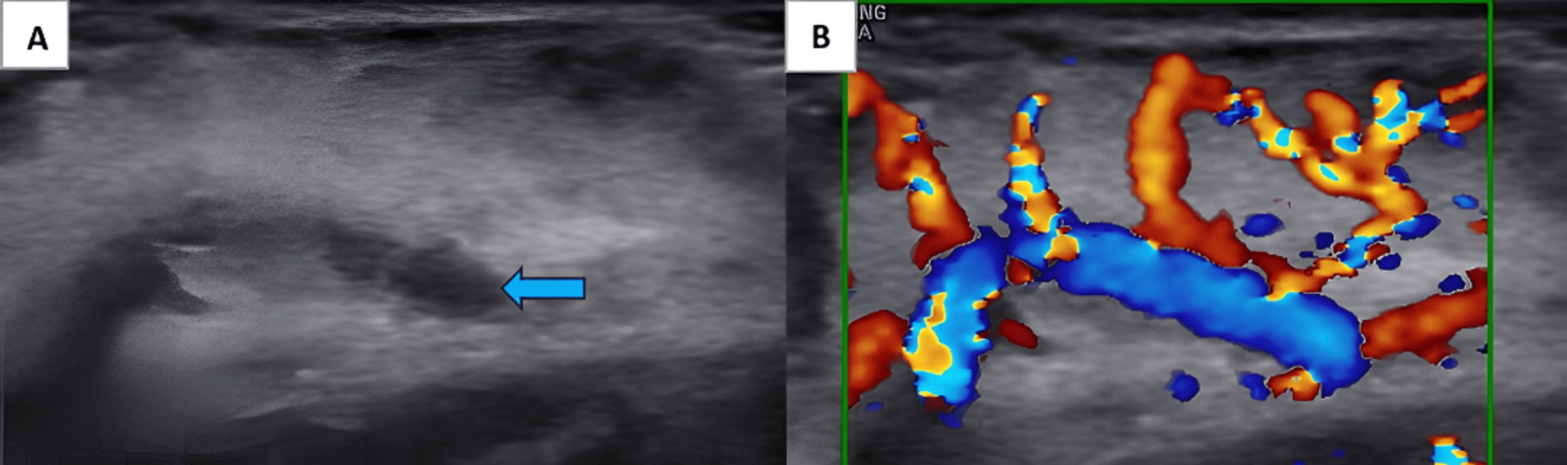
(A) B-mode USG showing predominantly hyperechoic lesion with hypoechoic tubular channels (blue arrow) within it and (B) color Doppler USG showing raised vascularity with feeding vessel noted within it USG: ultrasonography

B-mode ultrasonography using a high-frequency transducer revealed a well-circumscribed, heterogeneous, predominantly hyperechoic lesion with hypoechoic tubular channels noted over the dorsal aspect of the tongue on the right side. On color Doppler, it showed significantly raised vascularity with a feeding vessel noted within it (Figure 2).

**Figure 3:**
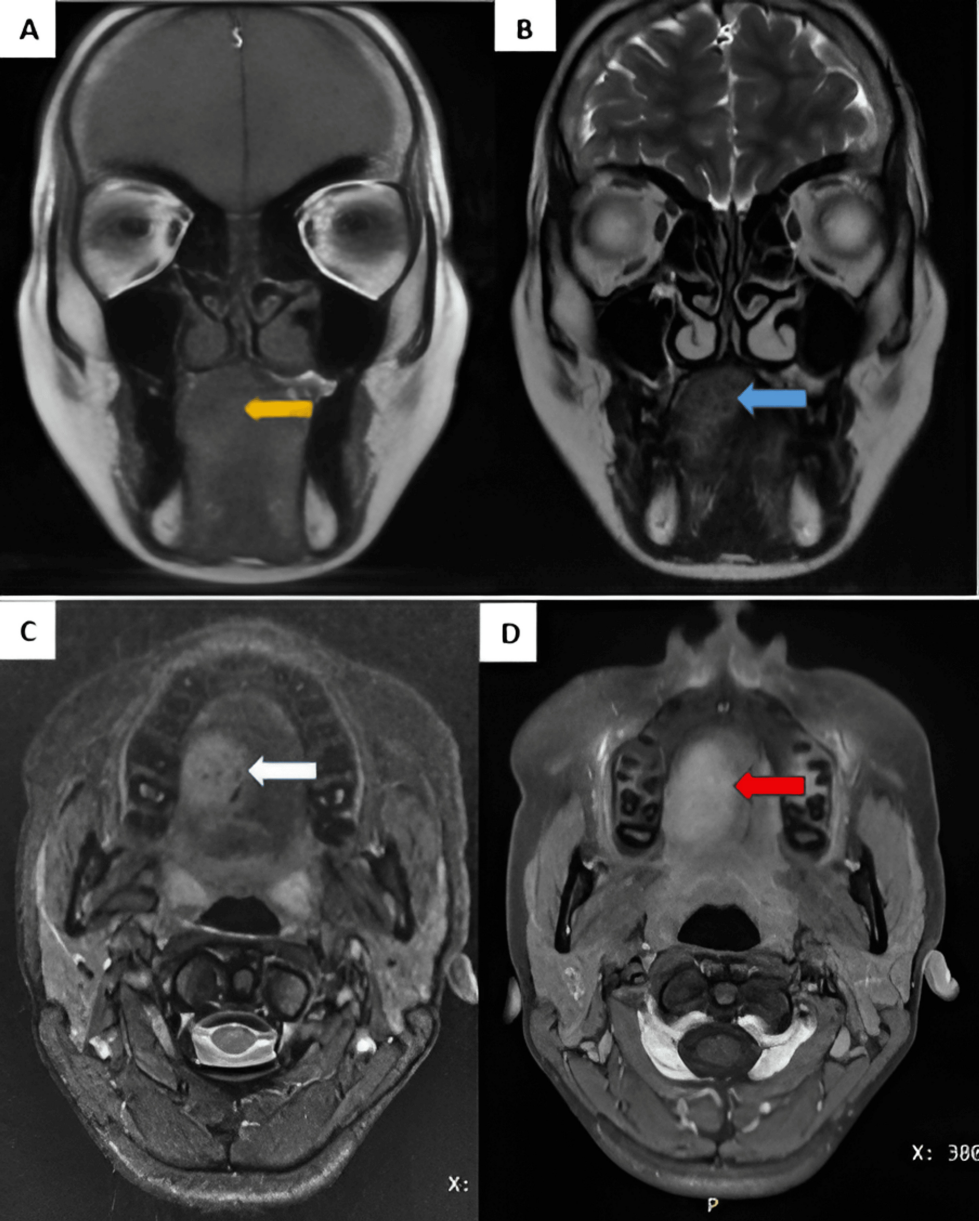
(A) Coronal T1WI, (B) coronal T2WI, (C) axial STIR, and (D) axial T1 + C A well-defined altered signal intensity lesion on the dorsal aspect of the tongue on the right side appearing T1WI isointense (yellow arrow) as compared to the muscle and T2WI (blue arrow)/STIR hyperintense, containing flow voids (white arrow), and showing homogeneous post-contrast enhancement (red arrow) T1WI: T1-weighted image, T2WI: T2-weighted image, STIR: short-tau inversion recovery, T1 + C: post-contrast T1-weighted image

1.5T MRI showed a well-defined altered signal intensity lesion of size approximately 2.7 × 1.9 × 2.5 cm (AP × TR × CC dimensions, respectively). It appeared isointense to the muscle on T1WI and hyperintense on T2/STIR involving the genioglossus and hyoglossus muscles of the tongue over the dorsal aspect on the right side, containing flow voids. On post-contrast (gadolinium) imaging, it showed homogenous enhancement. No adjacent bone involvement or extrinsic muscle infiltration was noted. These findings were consistent with intramuscular hemangioma (Figure 3).

**Figure 4:**
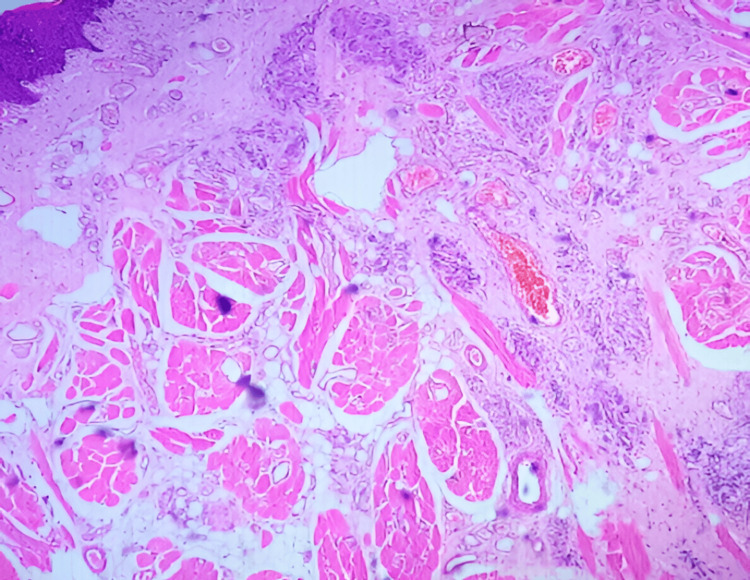
Histopathology revealing skeletal muscle bundles interspersed with numerous vascular channels of various calibers, lined by a layer of flattened endothelial cells, suggestive of cavernous-type intramuscular hemangioma

The patient underwent complete transoral excision. Histopathology revealed skeletal muscle bundles interspersed with numerous vascular channels of various calibers, lined by a layer of flattened endothelial cells without atypia or mitotic activity. Some vessels contained erythrocytes, and there was no evidence of thrombosis, necrosis, or malignancy. The lesion was lobulated and infiltrated between skeletal muscle fibers, while the overlying mucosa was intact with no dysplasia. These findings were consistent with a cavernous-type intramuscular hemangioma (Figure 4).

Complete resection of the lesion was done with clear margins. Peri- and postoperative status was uneventful. Follow-up on months 1, 2, and 6 showed no recurrence.

## Discussion

Intramuscular hemangioma (IMH) is a rare non-malignant vascular tumor arising from skeletal muscle. It accounts for less than 1% of all hemangiomas. Deep intramuscular location, slow progressive growth, and absence of characteristic features of the lesion result in late diagnosis and frequent misinterpretation as other benign soft tissue lesions [1].

IMH of the oral cavity is uncommon, with the tongue being a particularly unusual site of involvement. Unlike superficial hemangiomas, lingual IMH usually lacks mucosal discoloration, pulsation, or compressibility and often presents as a painless or mildly painful intramuscular swelling. Diagnostic difficulty arises because of the atypical clinical presentation of the lesion, which can delay appropriate management [2,3]. Many case reports have highlighted that lingual IMH may be clinically misdiagnosed as cystic hygroma, especially when presenting as a soft, non-pulsatile mass. Therefore, the need for careful radiological evaluation is needed in deep-seated tongue lesions [4].

For preoperative planning and diagnosis, imaging plays a central role. Ultrasonography may reveal a heterogeneous intramuscular lesion with variable internal vascularity on color Doppler imaging, although findings are often nonspecific. MRI is considered the imaging modality of choice because of its superior soft tissue contrast and multiplanar capability. Typical MRI features include an ill-defined or lobulated intramuscular mass that appears iso- to hypointense on T1-weighted images and markedly hyperintense on T2-weighted images, sometimes with internal septations, flow voids, or phleboliths corresponding to vascular channels [5,6]. These imaging characteristics help differentiate IMH from cystic, inflammatory, and non-vascular neoplastic lesions of the tongue.

There are three histological patterns: capillary, cavernous, or mixed types. The cavernous subtype is more commonly reported in adult lingual lesions [7,8]. The infiltrative growth pattern in muscle fibers determines the greater chance of recurrence, particularly in improper surgical excision [1].

Complete surgical excision remains the treatment of choice. However, due to the vascular nature and infiltrative margins of these lesions, surgery may be associated with significant intraoperative bleeding. Minimally invasive treatment options such as laser photocoagulation can be successfully done in selected cases, particularly when the lesion is large or in surgically challenging tongue hemangiomas [9]. Additionally, IMH is well recognized as a lesion that is easily misdiagnosed, reinforcing the importance of radiological awareness to prevent inappropriate management [10]. Recurrence after excision occurs in approximately 9%-28% of intramuscular hemangiomas, mainly due to incomplete resection of infiltrative lesions [1,2,5].

In conclusion, for deep-seated lingual masses, intramuscular hemangioma of the tongue is a rare but important differential diagnosis. Identification of characteristic ultrasound and MRI features followed by histopathological correlation is essential for accurate diagnosis, optimal treatment planning, and reduction of recurrence risk.

Differential diagnosis

Lymphatic Malformation (Cystic Hygroma)

Lymphatic malformation often presents as a soft, painless tongue swelling and may be confused with intramuscular hemangioma due to its slow growth and cystic appearance, particularly in deep lesions. On MRI, it appears hypointense on T1 and hyperintense on T2, with multiloculated cystic areas and no solid enhancement; septal or peripheral enhancement may be seen [4].

Venous Malformation

Venous malformation may mimic intramuscular hemangioma clinically and radiologically; however, venous malformations usually show slow-flow characteristics and lack a solid muscular component. On MRI, it appears iso- to slightly hyperintense on T1 and hyperintense on T2 signal with slow, homogeneous enhancement, and possible phleboliths; it lacks high-flow vascular characteristics of hemangiomas [1,10].

Benign Soft Tissue Tumors (Lipoma and Fibroma)

These lesions present as well-defined, painless tongue masses. Lipoma shows high signal on T1 and T2, suppressed on fat-saturated sequences. Fibroma shows low to intermediate signal on T1 and T2, with minimal enhancement. Both lack vascular flow and enhancement seen in intramuscular hemangiomas [2,5].

## Conclusions

Intramuscular hemangioma of the tongue is a non-malignant vascular tumor. It is difficult to diagnose as it arises from deep intramuscular tissue, and it shows a nonspecific clinical presentation. This case report emphasizes the role of B-mode ultrasonography, Doppler, and MRI. MRI plays an important role in lesion characterization, delineating its extent, and helps in guiding the management. The MRI features of a T2 hyperintense lesion with flow voids and an enhancing intramuscular lesion are key to establishing a reliable preoperative diagnosis. Complete transoral surgical removal of the lesion was performed, and the diagnosis was confirmed on histopathological examination. Early identification of this lesion with a multidisciplinary approach of clinical, radiological, and pathological diagnosis were essential to ensure optimal surgical treatment, thus preserving tongue function.
